# A preliminary study of optimal treatment response rates in patients undergoing hepatic arterial infusion chemotherapy combined with molecular targeting and immunotherapy

**DOI:** 10.3389/fimmu.2024.1303259

**Published:** 2024-04-10

**Authors:** Mei Li, Jun Liao, Li Wang, Tianye Lv, Qianfu Sun, Yan Xu, Zhi Guo, Manman Quan, Hao Qin, Haoyang Yu, Kai Zhang, Wenge Xing, Haipeng Yu

**Affiliations:** ^1^ Department of Interventional Therapy, Tianjin Medical University Cancer Institute & Hospital, National Clinical Research Center for Cancer, Tianjin, China; ^2^ Tianjin’s Clinical Research Center for Cancer, Tianjin Medical University Cancer Institute & Hospital, Tianjin, China; ^3^ Key Laboratory of Cancer Prevention and Therapy, Tianjin Medical University Cancer Institute & Hospital, Tianjin, China; ^4^ Norman Bethune Second Clinical Medical College, Jilin University, Changchun, China; ^5^ Department of Hepatobiliary Surgery, National Cancer Center/National Clinical Research Center for Cancer/Cancer Hospital, Langfang, China; ^6^ Chinese Academy of Medical Sciences (CAMS) and Peking Union Medical College (PUMC), Beijing, China

**Keywords:** targeted therapy, immunotherapy, FOLFOX-HAIC, surrogate endpoint, combination therapy, overall survival

## Abstract

**Objectives:**

This study aimed to examine the effectiveness of the best response rate (BRR) as a surrogate for overall survival (OS), using the modified Response Evaluation Criteria in Solid Tumors (mRECIST), in patients with unresectable hepatocellular carcinoma (HCC) undergoing hepatic arterial infusion chemotherapy (HAIC) with fluorouracil, leucovorin, and oxaliplatin (FOLFOX) combined with molecular targeting and immunotherapy.

**Methods:**

This study enrolled 111 consecutive patients who had complete imaging data. The median age of patients was 58 years (IQR 50.5-65.0). Among the patients, those with Barcelona Clinic Liver Cancer (BCLC) stage A, BCLC stage B, and BCLC stage C comprised 6.4%, 19.1%, and 73.6%, respectively. The optimal threshold of BRR can be determined using restricted cubic splines (RCS) and the rank sum statistics of maximum selection. Survival curves of patients in the high rating and low rating groups were plotted. We then used the change-in-estimate (CIE) method to filter out confounders and the inverse probability of treatment weighting (IPTW) to balance confounders between the two groups to assess the robustness of the results.

**Results:**

The median frequency of the combination treatment regimens administered in the overall population was 3 times (IQR 2.0-3.0). The optimal BRR truncation value calculated was −0.2. Based on this value, 77 patients were categorized as the low rating group and 34 as the high rating group. The differences in the OS between the high and low rating groups were statistically significant (7 months [95%CI 6.0-14.0] vs. 30 months [95%CI 30.0-]; p< 0.001). Using the absolute 10% cut-off value, the CIE method was used to screen out the following confounding factors affecting prognosis: successful conversion surgery, baseline tumor size, BCLC stage, serum total bilirubin level, number of interventional treatments, alpha-fetoprotein level, presence of inferior vena cava tumor thrombus, and partial thrombin activation time. The survival curve was then plotted again using IPTW for confounding factors, and it was found that the low rating group continued to have better OS than the high rating group. Finally, the relationship between BRR and baseline factors was analyzed, and inferior vena cava tumor thrombus and baseline tumor size correlated significantly with BRR.

**Conclusions:**

BRR can be used as a surrogate endpoint for OS in unresectable HCC patients undergoing FOLFOX-HAIC in combination with molecular targeting and immunotherapy. Thus, by calculating the BRR, the prognosis of HCC patients after combination therapy can be predicted. Inferior vena cava tumor thrombus and baseline tumor size were closely associated with the BRR.

## Introduction

HCC constitutes approximately 90% of all primary liver cancers and ranks fourth as the leading cause of cancer-related mortality. Although the incidence of virus-associated HCC has potentially reduced through vaccination and antiviral therapy, the incidence of HCC associated with other etiologies has escalated rapidly (1, 2). According to most clinical practice guidelines, patients with early HCC (BCLC stages 0 and A) should undergo excision, thermal ablation, or transplantation, while those with intermediate (BCLC stage B) and advanced (BCLC stage C) HCC should receive transarterial chemoembolization (TACE) or systemic therapy (3, 4). Nonetheless, the effectiveness of TACE for HCC with a substantial tumor burden is suboptimal. High-concentration drug chemotherapy without embolization can be administered through HAIC to treat localized tumors. Mounting evidence suggests that HAIC with fluorouracil, leucovorin, and oxaliplatin yields superior therapeutic outcomes and fewer adverse effects than TACE (5–7). In Asia, HAIC has been extensively employed as an alternative therapy to sorafenib in patients with advanced HCC (8, 9).

The latest developments in systemic therapy for advanced liver cancer have opened up fresh prospects for multimodal treatment of this disease. In the last decade, multi-kinase inhibitors aimed at tumor angiogenesis have been prescribed for advanced HCC (10–12). More recently, immune checkpoint inhibitors (ICI) that target the programmed cell death-1 (PD-1) pathway have emerged as the mainstream option for advanced HCC combination therapy, considering their favorable safety profile and promising objective response (13).

Recent studies have shown that for primary unresectable HCC, HAIC treatment combined with targeted therapy and immunotherapy can achieve a significant surgical conversion rate (14, 15). Although these findings are optimistic for individuals with unresectable HCC, it is important to note that not all patients may benefit from this combination therapy. In the realm of liver cancer research, the primary endpoint remains OS. However, to employ OS as an endpoint, several endpoint events must be documented, which could significantly amplify the intricacy of clinical research. Objective response rate (ORR) is now emerging as a crucial indicator for early evaluation of treatment efficacy (16–18). To our knowledge, no studies have explored whether the BRR can be used as a surrogate endpoint for OS in patients with HCC receiving combination therapy. Within this framework, this study aimed to investigate the effectiveness of BRR as a surrogate for OS based on the modified Response Evaluation Criteria in Solid Tumors (mRECIST) in HCC patients undergoing FOLFOX-HAIC therapy in conjunction with molecular targeting and immunotherapy.

## Materials and methods

### Eligibility of patients

This retrospective study followed the ethical principles of the Declaration of Helsinki and received approval from the Cancer Institute and Hospital Review Committee of Tianjin Medical University (bc2020099). Each participant was assigned a random number, and all identifying information was expunged to ensure anonymity (19). This study retrospectively collected clinicopathologic data and prognostic information of patients who underwent HAIC-FOLFOX combined with molecular targeting and immunotherapy at the Interventional Therapy Department of Tianjin Medical University Cancer Hospital between August 2019 and December 2021.

The inclusion criteria were as follows (1): diagnosis of initial unresectable liver cancer through multidisciplinary consultation; (2) clinical or pathological diagnosis of HCC; (3) Child-Pugh Grade A, Eastern Cooperative Oncology Group score ≤1, and routine laboratory tests indicating tolerance to the combined regimen; (4) no previous TACE, ablation, or other local treatment; (5) at least one course of HAIC combined with molecular targeting and immunotherapy received; and (6) complete imaging data available for evaluation.

The measurement of the maximum tumor diameter was conducted by two independent imaging doctors using dynamic contrast-enhanced computed tomography or magnetic resonance imaging, with neither examiner possessing any prior knowledge of the patient’s clinical data.

### Therapeutic procedure

Local anesthesia was induced in the patient, and the femoral artery was punctured by the Seldinger puncture technique. Digital subtraction angiography was used to display the anatomical characteristics of the abdominal cavity, superior Mesentery artery and hepatic artery, and to check the blood supply of the tumor site. A 2.7-F microcatheter is used in HAIC for implantation in the tumor-supplying artery. The FOLFOX regimen comprised oxaliplatin 85 mg/m^2^ infusion for 4 h, calcium folinate 400 mg/m^2^ infusion for 2-3 h, fluorouracil 400 mg/m^2^ injection once, and fluorouracil 1200 mg/m^2^ infusion for 23 h on the first day of treatment. HAIC is repeated every 4-6 weeks. If toxicity is intolerable, treatment may need to be interrupted or dosage adjusted.

Before or after the first HAIC treatment, the patients were administered anti-PD-1 antibodies intravenously every 3 weeks. For antiangiogenic therapy, the patients were administered 8 mg of lenvatinib orally once daily, sorafenib 200 mg twice daily, and apatinib 250 mg once daily. Please refer to our previously published 001 Research for detailed treatment of patients (14).

### Assessment of the treatment response

The evaluation of individual patient responses involved two independent imaging doctors who were blinded to other clinical data to mitigate potential bias. As per the mRECIST, BRR is characterized by the most substantial percentage decrease in the sum of the diameters of the target lesions as compared to the baseline target lesion diameters following multiple treatments (16, 18). A previous study on TACE therapy for liver cancer showed that ORR was associated with tumor burden. The study used a “6 + 12” score to define tumor burden, the sum of tumor size and number ≤ 6; the sum > 6 but ≤ 12; and the sum > 12 for low/intermediate/high tumor burden, respectively. For patients with low to moderate tumor burden, both the initial ORR and optimal ORR were prognostic indicators, while for patients with high tumor burden, only optimal ORR was prognostic (20). Our study primarily enrolled patients with medium to large liver cancer tumors, leading to the selection of the BRR as a prognostic indicator.

The OS is defined as the duration from the commencement of combination therapy until death from any cause or the date of the final evaluation. The progression-free survival (PFS), on the other hand, is determined by calculating the time from the initiation of combination therapy until either tumor progression is documented or death occurs, whichever comes first. The follow-up period ended in December 2022.

### Statistical analysis

Fisher’s exact test was used to analyze the categorical variables, and the results are expressed as numbers and percentages. Normally distributed data were analyzed using t-tests or variance analysis, while non-normally distributed data were analyzed using the rank sum test; these data are presented as medians and interquartile ranges (IQR). The Kaplan-Meier method was used for the estimation of survival.

In this study, the RCS method and the maximally selected rank statistics of the survminer package of R software were used to establish the optimal threshold of BRR. Restricted cubic splines is one of the common methods to fit the nonlinear relationship between independent and dependent variables (21). The patients were divided into two groups according to the BRR value, and the survival curves were plotted for each group. To reduce potential selection and confounding bias, we first screened out confounders using the CIE method and then balanced the inter-group confounders using the IPTW method to assess the robustness of the relationship between the BRR groups and OS. The CIE method, a data-driven independent variable screening method, is used to reduce the number of independent variables by eliminating the variables that have limited influence on the important independent variable effect in the multifactor regression model (22). IPTW takes the reciprocal probability of each observed value as the weight of the observed value to correct the estimation bias caused by missing data or biased sampling (23, 24). In this study, R software (version 4.2.1) was used for statistical analyses. The R packages used included tidyverse, survival, survminer, ggplot2, rcssci, and chest; p-values <0.05 were considered statistically significant.

## Results

This study enrolled 111 consecutive patients with complete imaging data who underwent at least one combination regimen (FOLFOX-HAIC combined with molecular targeting and immunotherapy). The median frequency of the combination treatment regimens administered in the overall population was three times.

**Figure 1:**
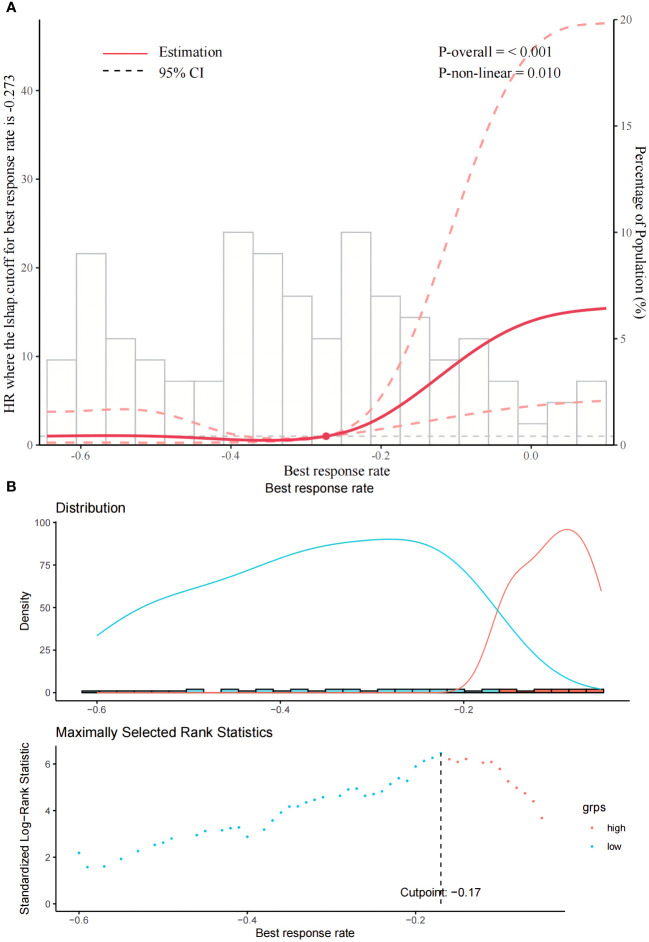
**(A, B)** present the schematics of the restricted cubic splines and rank sum statistics, respectively, to select the best response rate truncation values.

The BRR truncation value calculated using the RCS was −0.27 (**Figure 1A**), while that calculated using the survminer package was −0.17 (**Figure 1B**). Therefore, we considered the mid-value of the two, namely −0.2, as the final best truncation value of BRR. Those with a BRR <−0.2 were considered to have a high therapeutic response rate and were categorized as the low rating group (n = 77/111), whereas those with a BRR >−0.2 were considered to have a low therapeutic response rate and were categorized as the high rating group (n = 34/111). **Table 1** presents the baseline characteristics of the two groups of patients. Overall, the median age of patients was 58 years (IQR 50.5-65.0), with 85.6% being male. The most commonly used targeted drugs were lenvatinib (53.2%) and bevacizumab (38.7%), and most of the patients chose sintilimab (70.3%) as the immunotherapy agent. The primary etiology of HCC was hepatitis B virus (94.6%). The median lesion diameter was 8.9 cm (IQR 6.3-13.0). According to the BCLC system, 6.4%, 19.1%, and 73.6% of the cases in this study were categorized as stages A, B, and C, respectively. The tumor responses of the patients were shown in **Table 2**. On the basis of mRECIST criteria, the ORR of patients in the low rating group (78.0% vs 0, P < 0.001) was higher than that in the high rating group. The BRR of patients in the low rating group was lower than that in the high rating group (-0.4 vs -0.1, P < 0.001).

**Table 1:** Baseline demographic and clinical characteristics of high rating group and low rating group. .

	All patients	High rating group	Low rating group	p-value
	N=111	N=34	N=77	
Sex				1.000
Female	16 (14.4%)	5 (14.7%)	11 (14.3%)	
Male	95 (85.6%)	29 (85.3%)	66 (85.7%)	
Age	58.0 [50.5;65.0]	52.0 [45.0;63.8]	59.0 [54.0;65.0]	0.024
Old(≥60years)	45 (40.5%)	12 (35.3%)	33 (42.9%)	
Young(<60years)	66 (59.5%)	22 (64.7%)	44 (57.1%)	
Number of interventions	3.0 [2.0;3.0]	2.00 [2.0;3.0]	3.00 [2.0;3.0]	0.090
Successful conversion				0.002
No	73 (65.8%)	30 (88.2%)	43 (55.8%)	
Yes	38 (34.2%)	4 (11.8%)	34 (44.2%)	
Systemic therapy before intervention				0.143
No	92 (82.9%)	25 (73.5%)	67 (87.0%)	
Yes	19 (17.1%)	9 (26.5%)	10 (13.0%)	
Targeted drug				0.534
Apatinib	5 (4.5%)	1 (2.9%)	4 (5.2%)	
Bevacizumab	43 (38.7%)	12 (35.3%)	31 (40.3%)	
Lenvatinib	59 (53.2%)	21 (61.8%)	38 (49.4%)	
Sorafenib	4 (3.6%)	0	4 (5.2%)	
ICI				0.226
Atezolizumab	1 (0.9)	0	1 (1.3%)	
Camrelizumab	26 (23.4%)	11 (32.4%)	15 (19.5%)	
Pembrolizumab	1 (0.9%)	1 (2.9%)	0	
Sintilimab	78 (70.3%)	20 (58.8%)	58 (75.3%)	
Tislelizumab	2 (1.8%)	1 (2.9%)	1 (1.3%)	
Toripalimab	3 (2.7%)	1 (2.9%)	2 (2.6%)	
Hypertension				0.885
No	81 (73.0%)	24 (70.6%)	57 (74.0%)	
Yes	30 (27.0%)	10 (29.4%)	20 (26.0%)	
Diabetes				1.000
No	98 (88.3%)	30 (88.2%)	68 (88.3%)	
Yes	13 (11.7%)	4 (11.8%)	9 (11.7%)	
Heart disease				1.000
No	107 (96.4%)	33 (97.1%)	74 (96.1%)	
Yes	4 (3.6%)	1 (2.9%)	3 (3.9%)	
Smoking				0.220
No	79 (71.2%)	21 (61.8%)	58 (75.3%)	
Yes	32 (28.8%)	13 (38.2%)	19 (24.7%)	
Alcoholism				0.462
No	91 (82.0%)	26 (76.5%)	65 (84.4%)	
Yes	20 (18.0%)	8 (23.5%)	12 (15.6%)	
Etiology				1.000
Alchol	1 (0.9%)	0	1 (1.3%)	
HBV	105 (94.6%)	33 (97.1%)	72 (93.5%)	
HCV	5 (4.5%)	1 (2.9%)	4 (5.2%)	
Inferior vena cava invasion (IVCTT)				0.083
No	95 (85.6%)	26 (76.5%)	69 (89.6%)	
Yes	16 (14.4%)	8 (23.5%)	8 (10.4%)	
Vv classification				0.097
Vv0	95 (85.6%)	26 (76.5%)	69 (89.6%)	
Vv2	6 (5.4%)	4 (11.8%)	2 (2.6%)	
Vv3	10 (9.0%)	4 (11.8%)	6 (7.8%)	
Portal vein invasion (PVTT)				0.089
Distal	51 (45.9%)	11 (32.4%)	40 (51.9%)	
Proximal	60 (54.1%)	23 (67.6%)	37 (48.1%)	
Vp classification				0.305
Vp0	45 (40.5%)	10 (29.4%)	35 (45.5%)	
Vp2	6 (5.4%)	1 (2.9%)	5 (6.5%)	
Vp3	24 (21.6%)	9 (26.5%)	15 (19.5%)	
Vp4	36 (32.4%)	14 (41.2%)	22 (28.6%)	
BCLC stage				0.182
A	7 (6.4%)	1 (2.9%)	6 (7.9%)	
B	21 (19.1%)	4 (11.8%)	17 (22.4%)	
C	81 (73.6%)	28 (82.4%)	53 (69.7%)	
D	1 (0.9%)	1 (2.9%)	0	
Baseline tumor size	8.9 [6.3;13.0]	12.7 [8.7;15.0]	8.0 [5.4;11.0]	<0.001
WBC	5.6 [4.4;7.1]	5.7 [4.4;7.3]	5.4 [4.3;6.7]	0.630
PLT	169 [119;245]	201 [125;286]	160 [114;229]	0.128
PT	12.3 [11.8;13.0]	12.7 [12.1;13.0]	12.2 [11.7;13.1]	0.232
APTT	27.7 [25.5;30.0]	27.4 [25.7;30.6]	27.9 [25.2;30.0]	0.707
GLU	5.2 [4.5;5.9]	4.8 [4.3;5.9]	5.2 [4.7;5.9]	0.150
SCR	65.0 [58.0;76.0]	64.5 [58.5;69.8]	65.5 [58.0;76.8]	0.551
ALB	39.2 [35.9;42.0]	39.4 [36.2;42.2]	39.2 [35.5;41.9]	0.511
ALT	33.0 [21.0;49.0]	35.5 [24.8;50.2]	32.0 [19.0;49.0]	0.255
AST	50.0 [35.0;80.0]	62.5 [39.2;92.0]	49.0 [33.0;77.5]	0.068
TBIL	17.0 [11.8;22.7]	16.4 [12.2;23.0]	17.3 [11.4;22.4]	0.961
AFP				0.008
High(≥400 μg/L)	58 (53.7%)	24 (75.0%)	34 (44.7%)	
Low(<400 μg/L)	50 (46.3%)	8 (25.0%)	42 (55.3%)	

HBV, hepatitis B virus; HCV, hepatitis C virus; BCLC, Barcelona Clinic Liver Cancer; ICI, immune checkpoint inhibitors; WBC, white blood cell; PLT, platelet; PT, prothrombin time; APTT, activated partial thromboplastintime; GLU, glucose; SCR, serum creatinine; ALB, albumin; ALT, alanine aminotransferase; AST, aspartate transaminase;TBIL, total bilirubin; AFP, α-fetoprotein; CR, complete response; PD, progressive disease; PR, partial response; SD, stable disease;

Inferior vena cava tumor thrombus(IVCTT) were classified according to Japanese vv classification. Portal vein tumor thrombus(PVTT) were classified according to Japanese vp classification.

Vv classification: Vv0=no hepatic vein or inferior vena cava invasion, Vv1=peripheral hepatic vein invasion, Vv2=major hepatic vein invasion, Vv3=inferior vena cava invasion.

Vp classification: Vp0=no portal vein invasion, Vp1=distal invasion of the secondary portal vein, Vp2=portal vein secondary branch invasion, Vp3=invasion of the center and right branches of the portal vein, Vp4=invasion of main portal vein and above. Distal=Vp1+Vp2, Proximal=Vp3+Vp4.

In **Table 1**, “Yes” indicates the presence of the corresponding characteristic, while “No” indicates its absence. The characteristics include successful surgical conversion, receipt of systemic therapy before combined treatment, the presence of hypertension, diabetes, heart disease, smoking history, alcohol consumption history, the presence of inferior vena cava tumor thrombus, and the presence of portal vein tumor thrombus.

**Table 2:** Summary of best response by mRECIST criteria.

	All patients	High rating group	Low rating group	p-value
	*N=111*	*N=34*	*N=77*	
Maximum efficacy evaluation				<0.001
CR	1 (0.9%)	0	1 (1.3%)	
PD	6 (5.4%)	6 (17.6%)	0	
PR	59 (53.2%)	0	59 (76.6%)	
SD	45 (40.5%)	28 (82.4%)	17 (22.1%)	
ORR	60 (54.1%)	0	60 (78.0%)	<0.001
Best response rate	-0.3 [-0.5;-0.2]	-0.1 [-0.2;0.1]	-0.4 [-0.6;-0.3]	<0.001

CR, Complete response; PD, Progressive disease; PR, Partial response; SD, Stable disease.

**Figure 2:**
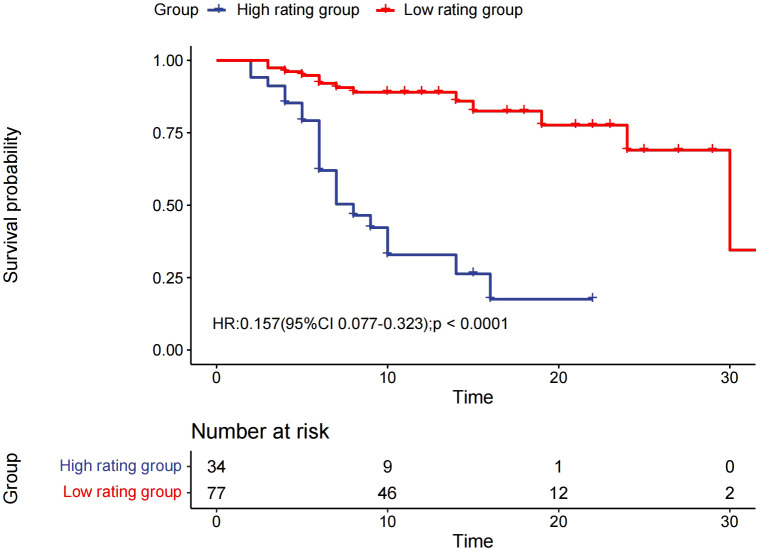
Kaplan–Meier curve for overall survival.

**Figure 3:**
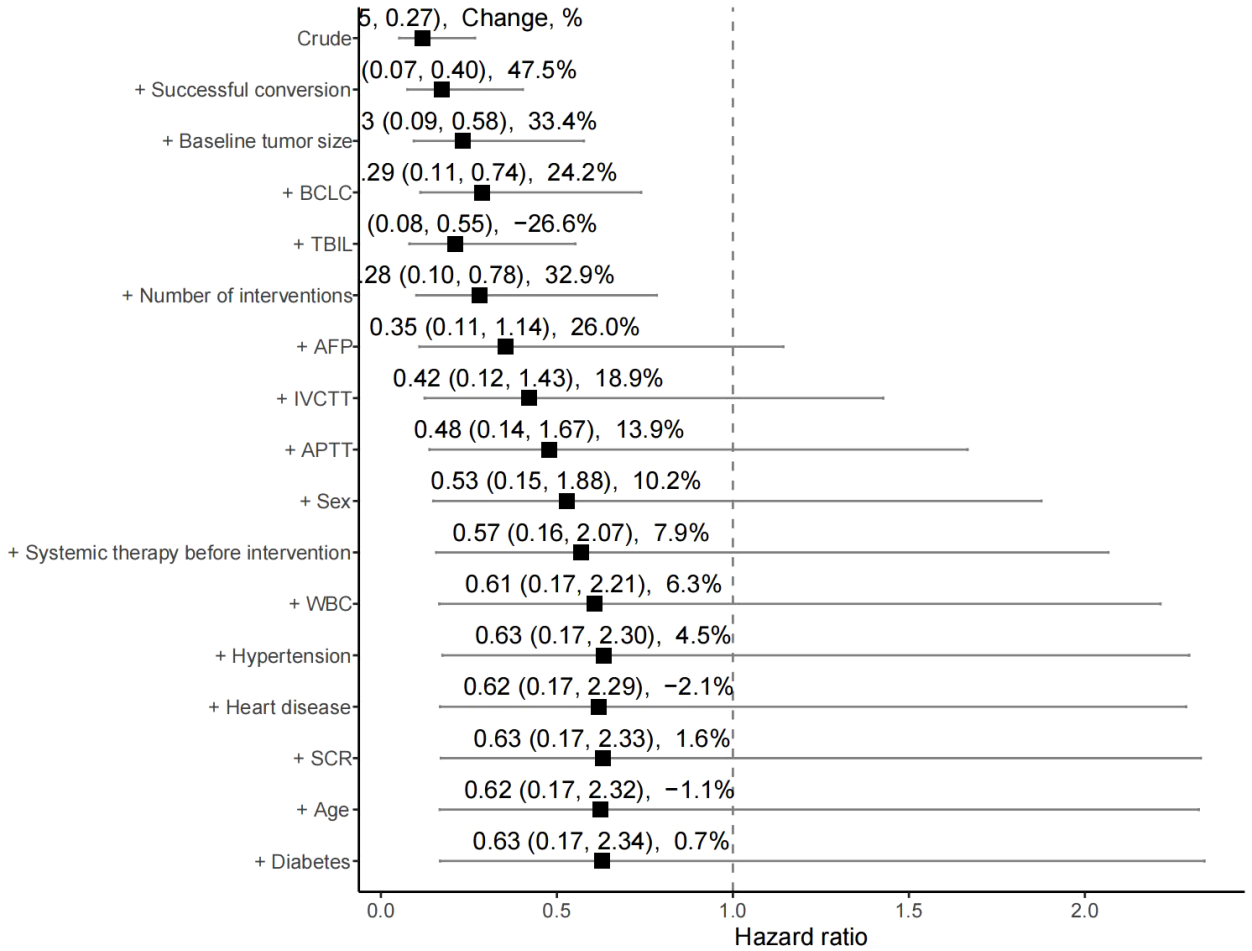
Change-in-estimate method for filtering variables. BCLC, Barcelona Clinic Liver Cancer; TBIL, Total bilirubin; AFP, α-fetoprotein; vv, Inferior vena cava tumor thrombus; APTT, Activated partial thromboplastintime; WBC, White blood cell; SCR, Serum creatinine.

**Figure 4:**
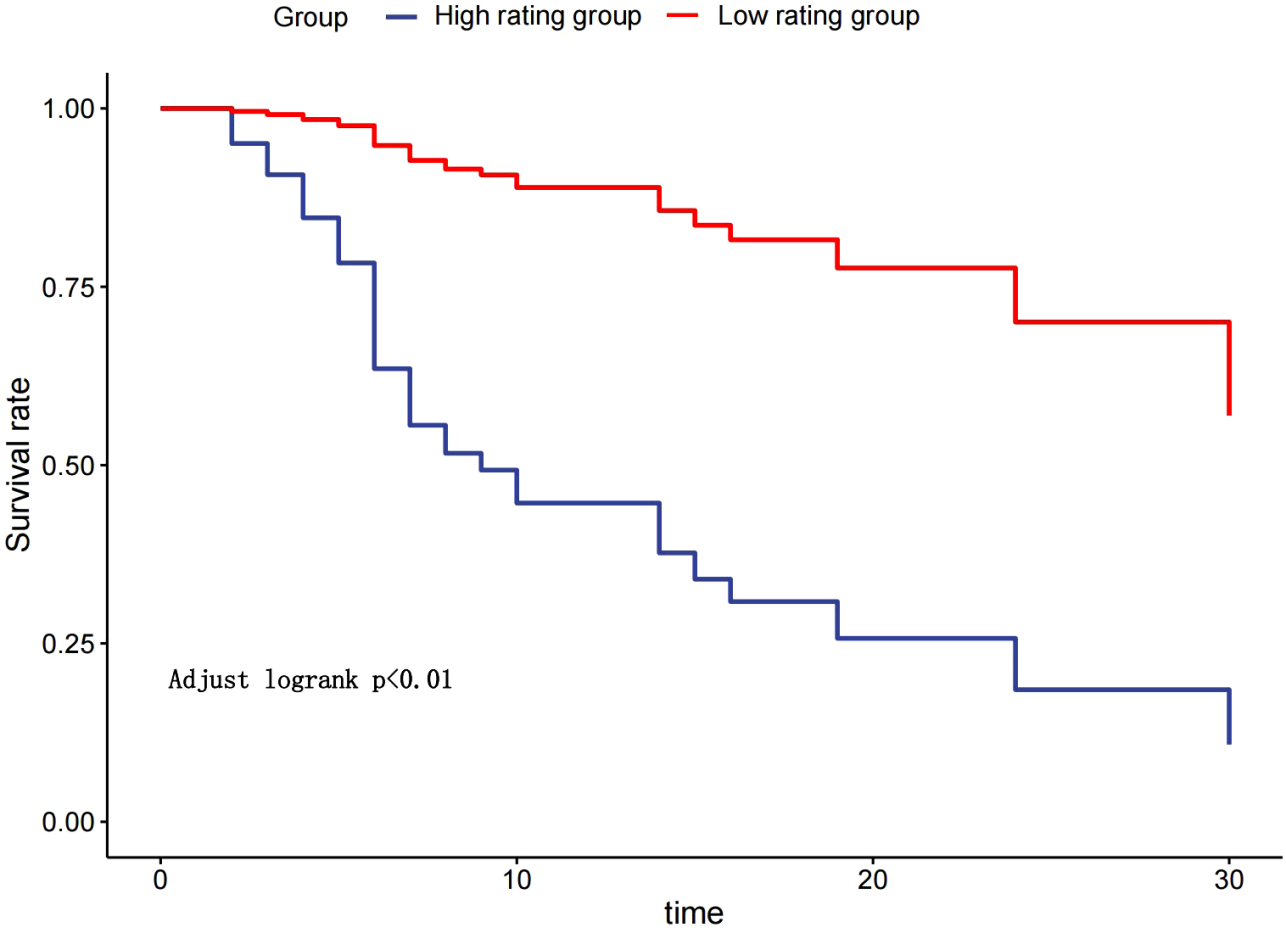
Kaplan–Meier curve after the inverse probability of treatment weighting.

The median OS was 7 months (95% CI 6.0–14.0) in the high rating group and 30 months (95% CI 30.0–) in the low rating group. The median PFS was 3 months (95% CI 3.0–5.0) in the high rating group and 19 months (95% CI 15.0–) in the low rating group. A statistically significant difference in the OS was observed between the two groups (p < 0.001) (**Figure 2**). Confounding factors screened by the CIE method included successful conversion surgery, baseline tumor size, BCLC stage, serum total bilirubin level, number of interventional treatments, alpha-fetoprotein (AFP) level, presence of inferior vena cava tumor thrombus, and partial thrombin activation time (**Figure 3**). We used the IPTW method to balance the confounding factors. The survival curves of the two groups were then redrawn, and log-rank tests were performed. The results showed that the low rating group continued to show significant improvements in the OS as compared to the high rating group (**Figure 4**) (p < 0.01).

**Figure 5:**
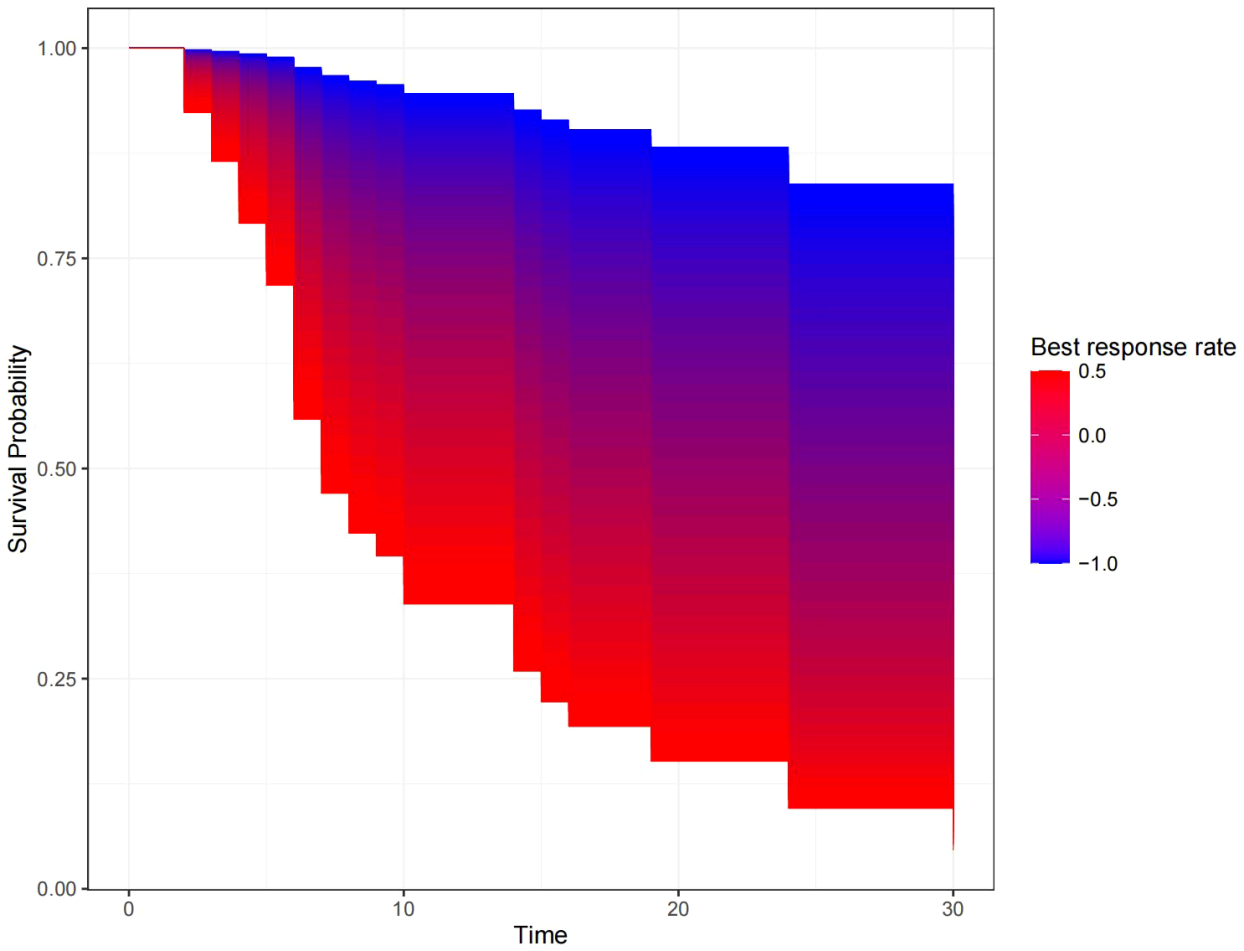
Kaplan–Meier curve with best response rate as a continuous variable.

**Figure 6:**
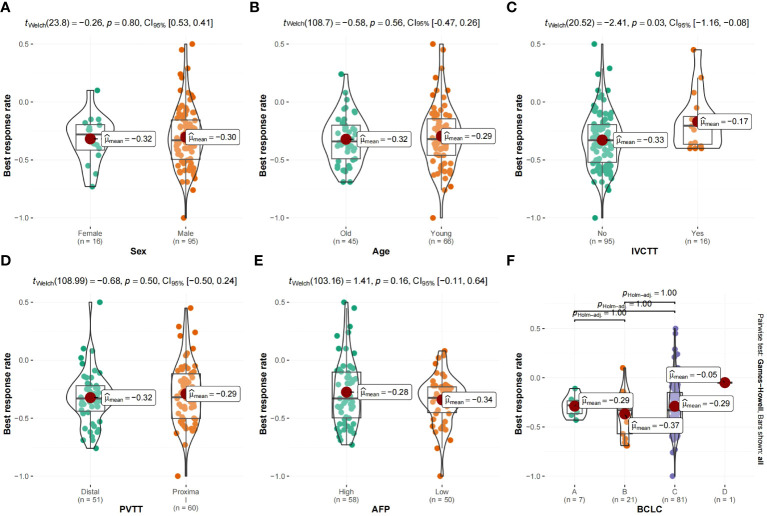
**(A–F)** Violin plot of categorical variables and best response rate. vv, Inferior vena cava tumor thrombus; vp, Portal vein tumor thrombus; AFP, α-fetoprotein; BCLC, Barcelona Clinic Liver Cancer.

**Figure 7:**
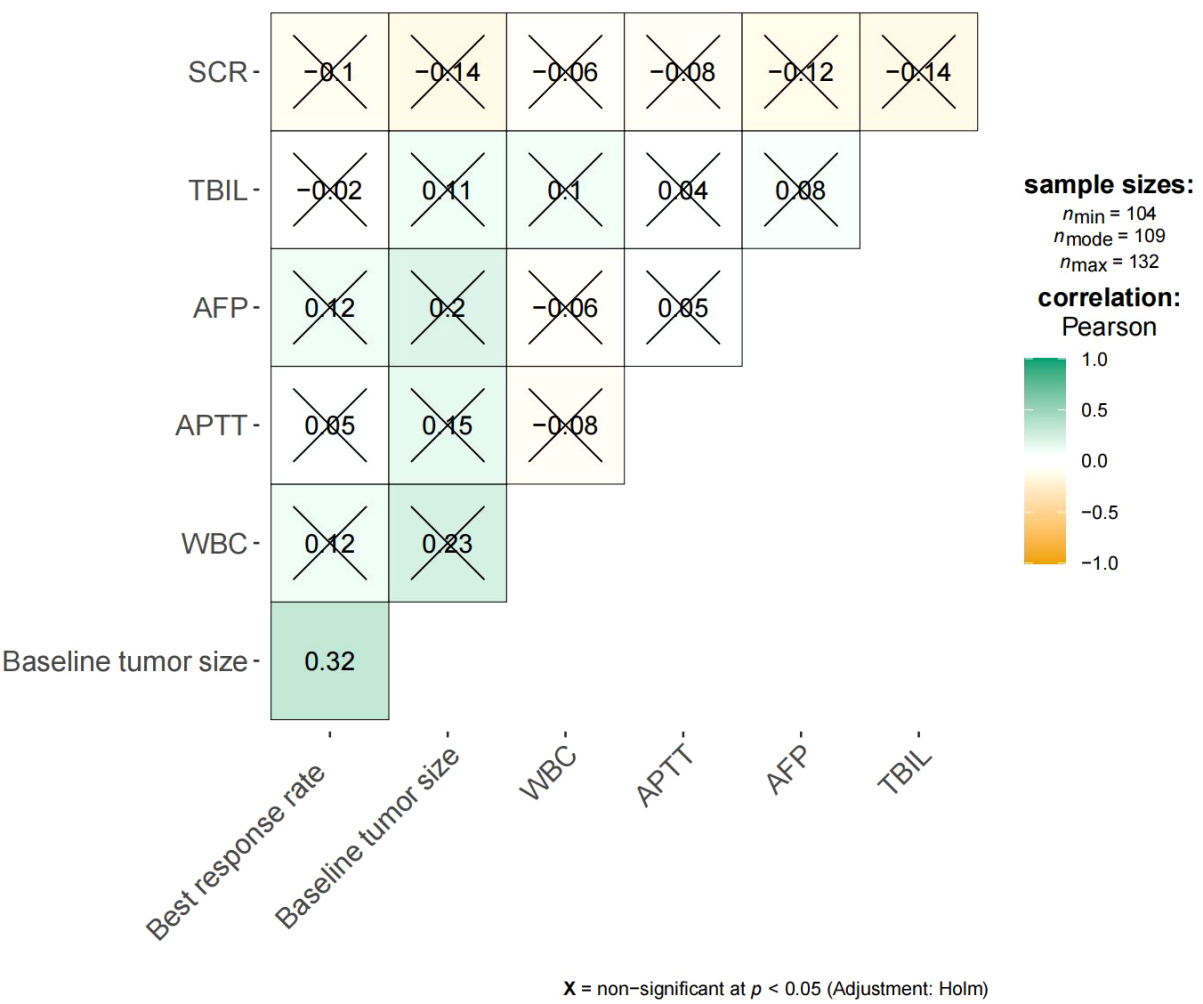
Heat map of continuous variables and best response rate. SCR, Serum creatinine; TBIL, Total bilirubin; AFP, α-fetoprotein; APTT, Activated partial thromboplastintime.

**Figure 5** shows the survival curve drawn with BRR as a continuity variable, wherein the greater the response rate, the longer the OS. We analyzed the correlation between BRR and other variables and found that inferior vena cava tumor thrombus (**Figure 6**) and baseline tumor size (**Figure 7**) significantly correlated with BRR. Inferior vena cava tumor thrombus negatively correlated with BRR, while baseline tumor size positively correlated with BRR (p < 0.05).

We gathered data on adverse events that occurred among patients receiving medications and HAIC therapy. All patients were evaluated for treatment-related adverse events using the CTCAE 4.0 grading system. Both groups exhibited similar frequencies of overall grade 1-2 and grade 3-4 adverse events (AEs), as reflected in **Table 3**. Specifically, the most common grade 1-2 AEs were pain, fatigue, and abnormal liver function, while the most frequent grade 3-4 AEs were fatigue, pain, and fever. Importantly, no patient died due to adverse events.

**Table 3:** Treatment-related adverse events.

Low rating group		High rating group		
	AE (1–2)	AE(3-4)	AE(1-2)	AE(3-4)
ALB reduce	3(4%)	0	2(6%)	1(3%)
ALT increased	11(14%)	0	5(15%)	1(3%)
Anemia	1(1%)	0	0	0
AST increased	8(10%)	0	5(15%)	1(3%)
BIL increased	4(5%)	1(1%)	3(9%)	1(3%)
Blood pressure increased	4(5%)	0	1(3%)	1(3%)
Diarrhea	4(5%)	2(3%)	2(6%)	1(3%)
Fatigue	12(16%)	9(12%)	4(12%)	7(21%)
Fever	2(3%)	19(25%)	2(6%)	7(21%)
Hand-foot skin reaction	2(3%)	0	1(3%)	0
Hypothyroidism	3(4%)	2(3%)	1(3%)	1(3%)
Nausea	5(6%)	4(5%)	3(9%)	2(6%)
Neutropenia	2(3%)	0	0	0
Pain	17(22%)	9(12%)	5(15%)	5(15%)
PLT reduce	6(8%)	1(1%)	2(6%)	0
Proteinuria	2(3%)	0	2(6%)	0
Rash	6(8%)	3(4%)	3(9%)	1(3%)
RCCEP	5(6%)	1(1%)	1(3%)	1(3%)
Vomiting	5(6%)	1(1%)	1(3%)	0

AE, Adverse event; ALB, Albumin; ALT, Alanine aminotransferase; AST, Aspartate transaminase; BIL, Bilirubin; PLT, Platelet; RCCEP, Reactive cutaneouscapillary endothelial proliferation;

These adverse events are graded according to CTCAE 4.0.

## Discussion

This study is the first to explore whether BRR can be used as an alternative endpoint to assess the efficacy of FOLFOX-HAIC therapy combined with molecular targeting and immunotherapy in patients with HCC. Both inferior vena cava tumor thrombus and baseline tumor size were important influencing factors for BRR.

Currently, the recommended initial therapy for advanced liver cancer involves administering a blend of molecular targeted drugs and ICI (25, 26). Targeted therapy combined with immunotherapy, as the mainstream treatment, has achieved a good local control rate and survival benefits in patients with advanced liver cancer; however, the ORR and OS remain unsatisfactory. Hence, researchers are trying to explore more effective combination treatment options (27, 28). Two studies conducted at our center suggested that patients with primary liver cancer undergoing HAIC in conjunction with immunotherapy and molecular targeted therapy exhibited a greater likelihood of undergoing surgical conversion and a notable survival advantage following surgery (14, 15).

Early and precise evaluation of tumor response to therapy is imperative for the optimal treatment of HCC (29). HCC can be diagnosed and monitored by imaging. The only validated non-invasive prognostic markers for HCC are tumor staging and AFP levels, and both have significant limitations in approximating tumor biology (30). Hence, formulating efficacious radiological criteria is crucial to identify HCC patients who would benefit from combination therapy. The criteria of the European Society for the Study of the Liver suggest ORR as a suitable alternative endpoint for assessing the efficacy of topical therapy (31–33). Several studies have demonstrated a correlation between objective response according to mRECIST and OS in patients (34, 35). Hence, it is a justifiable inference that the rate of tumor remission is associated with the prognosis. ORR is characterized by a decline of over 30% in the sum of the maximum diameters of the target lesion (The enhanced portion). The definition of BRR in this investigation is akin to that of ORR, albeit with a threshold of 20%, which may be relevant. The OS remains the principal outcome measure in clinical investigations on oncology and HCC. Nevertheless, it is imperative to ascertain a dependable secondary endpoint that can anticipate the OS. This study aimed to investigate the feasibility of utilizing BRR as a plausible substitute endpoint for assessing the efficacy of combination therapy in HCC. In clinical research, surrogate endpoints are employed as early indicators of treatment effectiveness instead of OS (36). Our study revealed noteworthy disparities in the OS between the high and low rating groups categorized based on the BRR truncation values. Notably, the majority of patients can undergo assessment for BRR after their third HAIC treatment session. However, it is imperative to acknowledge that a few patients may require supplementary therapy to attain a more significant tumor response. Nonetheless, the potential for hepatic injury linked to repeated surgeries renders this approach advantageous for only a restricted cohort of patients. Consequently, it is advisable that patients transition to an alternative treatment protocol if the optimal BRR is not attained following the third HAIC session. During the immunotherapy course with ICI, patients may exhibit atypical reaction patterns, including false progression, reaction separation, and delayed reaction. Notably, objective responses have been documented in non-small cell lung cancer patients up to 2 years following ICI treatment (37, 38). The median apparent time for FOLFOX-HAIC is approximately 3–4 treatment cycles (8, 39). This may explain why most patients achieve the BRR after undergoing three HAIC treatment sessions.

We screened and weighted factors that may affect the prognosis of HCC patients to verify the robustness of the relationship between BRR and OS. We included measures such as tumor characteristics (baseline tumor size, AFP level, and vascular invasion), serum bilirubin levels, blood-clotting parameters, and other liver function indicators. Additionally, age, underlying disease, sex, treatment, laboratory test values, and other indicators reflecting the basic state of the patient’s body were considered (40).

We finally analyzed the correlation between BRR and baseline features and found that BRR was associated with baseline tumor size and inferior vena cava tumor thrombus. The prognosis of HCC patients with inferior vena cava tumor thrombus is poor, and most of them eventually develop liver failure or cancer thrombus detachment shortly and die from pulmonary embolism or cardiac tamponade. There is no international consensus for treating HCC patients with inferior vena cava tumor thrombus (41, 42). Large liver cancer tumors at baseline imply late-stage cancers and poor liver function with a poor prognosis (25). Therefore, combination therapy should be recommended cautiously for HCC patients with large tumors or inferior vena cava tumor thrombus, as they may not benefit from this therapy.

The incidence of adverse events was comparable between the two groups, likely attributed to the absence of a significant disparity in the number of HAIC treatments administered. Nearly all patients encountered at least one adverse event. Specifically, 74% (82/111) of patients experienced at least one grade 3-4 adverse event, representing a notably higher incidence compared to previous studies (12, 15). This elevated frequency may be attributed to the inclusion of adverse events related to the perfusion therapy process, including fever and pain. These adverse events were manageable and did not significantly influence the prognosis of patients.

This retrospective study has certain limitations. First, its retrospective design resulted in inherent bias, including selection bias in patient inclusion and information bias in imaging data evaluation. Second, its retrospective and single-center nature may limit the generalizability of the findings to other cancer research centers. Thus, a prospective multicenter study is warranted to validate the results of this study.

In conclusion, this study shows that integrating FOLFOX-HAIC therapy with molecular targeting and immunotherapy can diminish the tumor burden effectively and expeditiously in several patients, leading to enhanced survival results. After three sessions of HAIC, patients with liver cancer undergoing combination therapy can assess their BRR to ascertain the potential benefits of the treatment.

## Data availability statement

The raw data supporting the conclusions of this article will be made available by the authors, without undue reservation.

## Ethics statement

The studies involving humans were approved by Tianjin Cancer Hospital Medical Ethics Committee. The studies were conducted in accordance with the local legislation and institutional requirements. Written informed consent for participation was not required from the participants or the participants’ legal guardians/next of kin in accordance with the national legislation and institutional requirements.

## Author contributions

ML: Writing – original draft, Conceptualization, Data curation. JL: Writing – review & editing. LW: Writing – review & editing. TL: Conceptualization, Data curation, Writing – review & editing. QS: Investigation, Software, Writing – review & editing. YX: Software, Conceptualization, Writing – review & editing. ZG: Supervision, Data curation, Writing – review & editing. MQ: Validation, Methodology, Writing – review & editing. HQ: Project administration, Methodology, Writing – review & editing. KZ: Data curation, Supervision, Writing – review & editing. WX: Resources, Funding acquisition, Writing – review & editing. HPY: Funding acquisition, Resources, Writing – review & editing. HYY: Data curation, Conceptualization, Formal analysis, Validation, Investigation, Funding acquisition, Software, Writing – original draft.
